# Molecular determinants of resistance in *Escherichia coli*-ST131 isolated blood stream infections

**DOI:** 10.1186/1471-2334-13-S1-O15

**Published:** 2013-12-16

**Authors:** Adriana Hristea, Raluca Jipa, Ioana D Olaru, Cristina Popescu, Victoria Aramă, Ruxandra Moroti, Oana Streinu-Cercel

**Affiliations:** 1 Carol Davila University of Medicine and Pharmacy, Bucharest, Romania; 2 National Institute for Infectious Diseases “Prof. Dr. Matei Balş”, Bucharest, Romania

## Background

During the past years an *E coli* clonal lineage ST131 has emerged explosively, causing predominantly community-onset antimicrobial resistant infections. *E coli* ST131 has been shown to harbor a number of virulence and resistance genes and is now recognized for its ability to cause potentially severe infections in many parts of the developing world, implying public health measures in attempt to control infection.

## Methods

Eighty-five drug-resistant *E coli* isolates from patients with blood stream infections (BSI) were screened by a ST131-clone allele-specific PCR for the *pabB* gene and analyzed for genes encoding ESBL by PCR and sequencing. Integrons were detected by PCR targeting the integrase gene. Gene cassette PCR was conducted on all class 1 integrase-positive isolates. BSI were classified according to the setting of infection: community acquired (CA), healthcare-associated (HCA), and hospital acquired (HA).

## Results

The sources of *E coli* BSI, were: urinary in 50 (59%), gastrointestinal in 9 (11%), respiratory in 4 (5%), other (including skin and soft tissue) in 3 (3%), unknown in 19 (22%) patients. By *pabB* PCR, we identified 22 (26%) *E coli* isolates belonging to the O25b-ST131 clonal lineage. Among 22 ST131 *E coli* isolates, 6 (27%) were from CA, 11 (50%) were from HCA, and 5 (23%) were from HA infections, while among 61 non-ST 131 *E coli* isolates, 30 (49%) were from CA, 14 (23%) were from HCA, and 17 (28%) from HA infections (p=0.053). ESBL genes were found in 20 (91%) ST131 and 13 (21%) non-ST131 *E coli* isolates (p<0.0001, OR=38.46 [95%CI: 7.95-186.06]). The ST131 isolates carried *bla*_CTX-M_ in 18 (82%), *bla*_TEM-1_ in 6 (27%) and *bla*_OXA-1_ in 18 (82%). Class I integrons were identified in 11 (50%) ST131-producing *E coli* strains and 36 (57%) non-ST131 *E coli* strains (p=0.623, OR=0.750 [95%CI: 0.283-1.9850]). Resistance to trimethoprim/sulfamethoxazole was found in 11 (50%) ST131 *E coli* isolates and in 32 (59%) non-ST131 *E coli* strains (p=0.610), while resistance to both fluoroquinolones and aminoglycosides was identified in 18 (82%) ST131 *E coli* isolates compared to 10 (16%) non-ST131 *E coli* strains (p<0.001). Among patients with BSI caused by resistant *E coli*, the in-hospital mortality was similar in patients infected with strains carrying the ST131 clonal lineage (3; 14%) versus non ST131 (10; 17%) (p=0.921).

## Conclusion

In our study group, BSI caused by *E coli* ST131 were most likely to be hospital rather than community associated. We found more ESBL genes in ST131 *E coli* isolates, while class I integrons were present in both groups in similar rates.

